# Systematic comparison of the statistical operating characteristics of various Phase I oncology designs

**DOI:** 10.1016/j.conctc.2016.11.006

**Published:** 2016-11-24

**Authors:** Revathi Ananthakrishnan, Stephanie Green, Mark Chang, Gheorghe Doros, Joseph Massaro, Michael LaValley

**Affiliations:** a Department of Biostatistics, Boston University, 801 Massachusetts Avenue 3rd Floor, Boston, MA 02118, USA; b Pfizer Oncology, 445 Eastern Point Road, Groton, CT 06340, USA

**Keywords:** Phase 1 designs, Oncology, Dose finding, Rule-based designs, Model-based designs, Accuracy of MTD selection, DLT, dose limiting toxicity, MTD, maximum tolerated dose, mTPI design, modified toxicity probability interval design, TEQR design, toxicity equivalence range design, BOIN design, Bayesian optimal interval design, CRM, continual reassessment method, EWOC design, escalation with overdose control design

## Abstract

Dose finding Phase I oncology designs can be broadly categorized as rule based, such as the 3 + 3 and the accelerated titration designs, or model based, such as the CRM and Eff-Tox designs. This paper systematically reviews and compares through simulations several statistical operating characteristics, including the accuracy of maximum tolerated dose (MTD) selection, the percentage of patients assigned to the MTD, over-dosing, under-dosing, and the trial dose-limiting toxicity (DLT) rate, of eleven rule-based and model-based Phase I oncology designs that target or pre-specify a DLT rate of ∼0.2, for three sets of true DLT probabilities. These DLT probabilities are generated at common dosages from specific linear, logistic, and log-logistic dose-toxicity curves. We find that all the designs examined select the MTD much more accurately when there is a clear separation between the true DLT rate at the MTD and the rates at the dose level immediately above and below it, such as for the DLT rates generated using the chosen logistic dose-toxicity curve; the separations in these true DLT rates depend, in turn, not only on the functional form of the dose-toxicity curve but also on the investigated dose levels and the parameter set-up. The model based mTPI, TEQR, BOIN, CRM and EWOC designs perform well and assign the greatest percentages of patients to the MTD, and also have a reasonably high probability of picking the true MTD across the three dose-toxicity curves examined. Among the rule-based designs studied, the 5 + 5 a design picks the MTD as accurately as the model based designs for the true DLT rates generated using the chosen log-logistic and linear dose-toxicity curves, but requires enrolling a higher number of patients than the other designs. We also find that it is critical to pick a design that is aligned with the true DLT rate of interest. Further, we note that Phase I trials are very small in general and hence may not provide accurate estimates of the MTD. Thus our work provides a map for planning Phase I oncology trials or developing new ones.

## 1 Introduction

Phase I trials of a new anti-cancer drug are usually single arm, open label studies conducted on a small number (10s) of cancer patients, many of whom do not respond any longer to the standard treatment. Due to the toxic nature of many anti-cancer drugs as well as due to ethical reasons, cancer patients are enrolled in Phase I oncology trials, as opposed to the healthy volunteers used in Phase I trials in other therapeutic areas.

The main aim of a Phase I oncology trial is to investigate and understand the toxic properties (safety) of the new anti-cancer drug; the drug's efficacy is not traditionally the focus, although the drug's efficacy is often observed and monitored by the oncologist. With regard to safety, the trial helps investigators determine the right dose and dosing interval as well as the best route of administration of the new drug. In order to determine the right dose, an endpoint such as Phase 1 dose limiting toxicities (DLTs) in the first cycle is often considered.

For each dose finding Phase I trial, a set of pre-defined adverse events, typically only those possibly related to taking the study drug, constitutes the DLTs for that trial. Patients are traditionally monitored for DLTs during the first cycle of administration of the new anti-cancer drug; however, more recent trials may monitor DLTs for a longer period and may include toxicities in the DLT definition that are not included in the conventional definition of DLTs [1]. The starting dose in these dose finding trials is usually a very conservative dose based on animal studies of the drug, and the subsequent increasing doses to be administered are pre-specified. The number of patients with DLTs in each dose level is used to determine the Maximum Tolerated Dose (MTD). For a single anti-cancer drug being tested, the MTD is usually the highest dose level at which the observed DLT rate is equal to or below a specified percent. Phase II patients are generally dosed at the MTD determined in the corresponding Phase I trial. The above method for MTD selection is more applicable to cytotoxic agents where the toxicity and efficacy are assumed to increase monotonically with dose than to some modern molecularly targeted therapies where the MTD may not be reached even at higher doses due to their low toxicity; in such cases, another appropriate dosing endpoint may need to be considered such as the dose at which the key pharmacokinetic and pharmacodynamics parameters are optimal [1], [2].

Dose finding Phase I oncology designs can be broadly categorized [3], [4], [5], [6] as rule based (such as the 3 + 3 design) or model based (such as the CRM [7] and Eff-Tox designs [8]). The 3 + 3 design has been the workhorse dose finding design for Phase I oncology trials for a long time. It is still commonly used due to its simplicity and ease of implementation. However, depending on the target DLT rate of interest, it can be slow and inaccurate in estimating the MTD and can lead to a large portion of patients receiving sub-therapeutic doses that do not produce any clinically meaningful response [9]. Hence, other designs, including model-based designs, have been explored in recent years [10], [11], [12].

The establishment of the MTD for various Phase 1 oncology designs is the main focus of this paper. In this work, we explore extensions of the 3 + 3 design as well as the model based mTPI [13], TEQR [14], BOIN [15], CRM [7], [3] and EWOC [16], [17] designs and compare their performance. There is no unique criterion to evaluate these designs since the performance of each design depends on the true DLT probability at each dose and the target DLT rate of the design. Hence, we systematically compare several statistical operating characteristics for the true DLT rates generated at the same doses by three different dose-toxicity curves. In addition, we explore the effect of starting the trial at different dose levels below the true MTD on the accuracy of MTD selection in these designs. The 3 + 3 design and its extensions we consider target a DLT rate of ∼0.2, and we specify a target DLT rate of 0.2 for the model based designs we consider. Although the results in this paper focus on a target DLT rate of 0.2, we explain in the discussion section the implications of targeting other DLT rates such as 0.1 and 0.33 with the A + B designs considered and discuss other A + B designs that target these rates. We also study the performance of the model based designs considered when the target DLT rate specified is 0.1 and 0.33. In contrast to previous works that compare a limited number of specific designs [18], our comprehensive comparison across several designs should serve as a practical aid in applying these Phase I oncology designs or in developing new ones.

## 2 Methods

### 2.1 Rule based designs

We consider the 3 + 3 design, which targets a DLT rate of ∼0.2 [19], as well as its various extensions that target a DLT rate of ∼0.2. We also include the simple accelerated titration design and the 3 + 3+3 design in our study (Table 1) [20], [21], [22]. We then investigate several of their statistical operating characteristics, such as the accuracy of MTD selection among others. The formal definition of the MTD is that it is the dose for which Probability(DLT|dose = d) = target probability.

**Table 1:** Designs investigated that are extensions of the 3 + 3 design that allow only escalation.

Design	Assignment rule	Ways to escalate	Approximate range for toxicity rate targeted by the design (Table 4.1, Chapter 4, Ting, 2006 [30]; Storer, 2001 [19])
3 + 3	If 0 out of 3 enrolled patients have a DLT, then escalate to the next dose level and enroll 3 more; if 1 out of 3 patients has a DLT, then add 3 more patients at the same dose level; if 2 or more patients out of 3 or 6 patients experience a DLT, then stop the trial. The MTD is one dose level below.	0/3 = 0% or 1/6 = 16.7% i.e. can escalate if we observe 0 DLTs out of 3 patients, or 1 DLT out of 6 patients	0.17<Γ < 0.26 or 0.2<Γ < 0.25
2 + 4	If 0 out of 2 enrolled patients have a DLT, then escalate to the next dose level and enroll 2 more; if 1 out of 2 patients has a DLT, then add 4 more patients at the same dose level; if 2 or more patients out of 2 or 6 patients experience a DLT, then stop the trial. The MTD is one dose level below.	0/2 = 0% or 1/6 = 16.7% i.e. can escalate if we observe 0 DLTs out of 2 patients, or 1 DLT out of 6 patients	0.17<Γ < 0.26
4 + 4 a	If 0 out of 4 enrolled patients have a DLT, then escalate to the next dose level and enroll 4 more; if 1 or 2 out of 4 patients have a DLT, then add 4 more patients at the same dose level; if 3 or more patients out of 4 or 8 experience a DLT, then stop the trial. The MTD is one dose level below.	0/4 = 0% or 1/8 = 12.5% or 2/8 = 25% i.e. can escalate if we observe 0 DLTs out of 4 patients, or 1 DLT out of 8 patients, or 2 DLTs out of 8 patients	0.25<Γ < 0.31
5 + 5 a	If 0 out of 5 enrolled patients have a DLT, then escalate to the next dose level and enroll 5 more; if 1 or 2 out of 5 patients have a DLT, then add 5 more patients at the same dose level; if 3 or more patients out of 5 or 10 experience a DLT, then stop the trial. The MTD is one dose level below.	0/5 = 0% or 1/10 = 10% or 2/10 = 20% i.e. can escalate if we observe 0 DLTs out of 5 patients, or 1 DLT out of 10 patients, or 2 DLTs out of 10 patients	0.2<Γ < 0.25
3 + 3+3	If 0 out of 3 enrolled patients have a DLT, then escalate to the next dose level and enroll 3 more; if 1 out of 3 patients has a DLT, then add 3 more patients at the same dose level; if 2 out of 6 patients have a DLT then add 3 more patients at the same dose level; if 2 or more patients out of 3 patients experience a DLT or 3 or more out of 6 or 9 patients experience a DLT, then stop the trial. The MTD is one dose level below.	0/3 = 0% or 1/6 = 16.7% or 2/9 = 22.2% i.e. can escalate if we observe 0 DLTs out of 3 patients, or 1 DLT out of 6 patients, or 2 DLTs out of 9 patients	
Simple Accelerated Titration Design	Successively assign a single patient at each dose level until the patient has a DLT. Then switch to the 3 + 3 design (i.e. add 2 more patients to the dose level at which a DLT is first seen and then follow the rules of the 3 + 3 design).		

The table above provides the rules for the escalation only designs but we also allow de-escalation in the 3 + 3, 2 + 4, 4 + 4 a, and 5 + 5 a designs and follow the algorithm described in the methods section. The designs that also allow de-escalation will target a slightly lower DLT rate than their counterparts that allow only escalation. One method to estimate the approximate target DLT rate of each design that also allows de-escalation is to run simulations for each design using several different dose-toxicity curves and then perform the following calculation: one needs to compute the sum of the product of the true DLT rate at each dose and the probability that that dose is selected as the MTD from simulations for each scenario and then find the average of this value across the various scenarios (dose-toxicity curves). Based on our results for the logistic, log-logistic and linear dose-toxicity curves in Table 3, Table 4, Table 5, we find that the approximate target DLT rate of the 3 + 3 design with de-escalation is 0.17, of the 2 + 4 design with de-escalation is 0.18, of the 4 + 4 a design with de-escalation is 0.21 (which is why we also included the 4 + 4 a design, even though its target DLT rate for the escalation only case is a little higher than 0.2), and of the 5 + 5 a design with de-escalation is 0.17. The 3 + 3+3 design targets an approximate DLT rate of 0.21.

For the A + B designs [23] that allow only escalation, the algorithm that we follow is [21]:

1) If out of A patients assigned to dose level i, the number of DLTs observed is ≤x, then assign A patients to dose level i+1.
2) If the number of DLTs observed out of A patients at dose level i is >x and <y, then assign B more patients to dose level i. If out of A + B patients, the number of DLTs observed is ≤z, then add A patients to dose level i+1. Otherwise stop the trial.
3) If the number of DLTs observed out of A patients at dose level i is ≥y, then stop the trial.

We then estimate the MTD to be the dose level immediately below the last dose level examined. For the standard 3 + 3 design (Table 1), which is a special case of the general A + B design, this implies that the MTD is estimated to be the highest dose in which fewer than 33% of patients experience a DLT.

For the A + B designs that also allow de-escalation, the algorithm that we follow is:

1) Implement the rules given above for the corresponding escalation only design and let i be the dose level where the number of DLTs exceeds that allowed by the design. Then, ensure that A + B patients have been dosed at dose level i-1. If yes, dose level i-1 is estimated to be the MTD.
2) If not, add B more patients at dose level i-1.
  a) If out of the A + B patients at dose level i-1, the number of DLTs observed is ≤z, then dose level i-1 is estimated to be the MTD even if A + B patients have not been dosed at dose level i-2.
  b) If out of the A + B patients at dose level i-1, the number of DLTs observed is >z and A + B patients have been dosed at dose level i-2, then dose level i-2 is estimated to be the MTD. If A + B patients have not been dosed at dose level i-2, then add B more patients and continue the process.

For the 3 + 3 design with de-escalation, the MTD is estimated to be the highest dose in which fewer than 33% of patients experience a DLT, and in which at least six participants have been treated with the study drug.

**Fig. 1:**
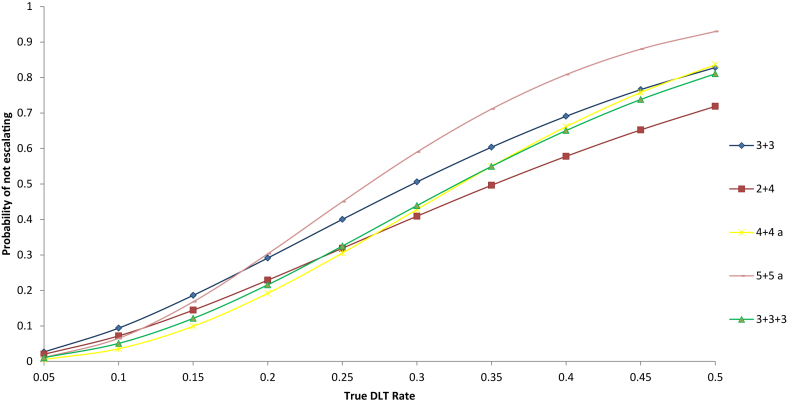
Depicts the probability of not escalating at each step for different true DLT rates for the escalation only designs considered that are extensions of the 3 + 3 design and that target a DLT rate of ∼0.2. These probabilities are derived analytically based on the decision rules of each design as given in Table 1.

For the rule-based designs where no de-escalation is allowed, Table 1 describes the dose finding rules; the specific x, y, and z for each A + B design can be determined based on the description of these designs in Table 1. To provide a preliminary idea of the properties of these designs, we depict in Fig. 1 the probability of not escalating for a single step for various true DLT rates for the escalation only designs considered. For example, for the 3 + 3 design that allows only escalation, we can escalate at each step or dose level if 1) 0 out of 3 patients experience a DLT or if 2) 1 out of 6 patients experiences a DLT; the probability of escalating at each step or dose level is q^**3**^+**3**pq^**5**^ and not escalating at each step is **3**p^**2**^q + p^**3**^+**9**p^**2**^q^**4**^+**9**p^**3**^q^**3**^+**3**p^**4**^q^**2**^, where p is the probability of experiencing a DLT at the current dose level and q = 1-p. Using these two probabilities and extending the framework to any number of steps, we can then calculate analytically the probability of selecting any dose level as the MTD for the 3 + 3 as well as other A + B designs that allow only escalation (see Lin, 2001 [24] and Appendix Table 1). This reference [24] also provides analytic formulae for the probability of MTD selection for the 3 + 3 and other A + B designs that allow de-escalation as well.

### 2.2 Model based designs or designs that allow specification of the target DLT rate

In terms of model-based designs, we consider the Modified Toxicity Probability Interval (mTPI), Toxicity Equivalence Range (TEQR), Bayesian Optimal Interval Design (BOIN), Continual Reassessment Method (CRM) and Escalation with Overdose Control (EWOC) designs and explore their statistical operating characteristics. For these designs, we can choose the DLT rate that each design will target; we specify a target DLT rate of 0.2 for all of them, in order to compare their performance with the performance of the 3 + 3 design and its extensions that target a DLT rate of ∼0.2. Note that although the TEQR design is not a model based design, it allows the specification of the target DLT rate.

The mTPI design is described in detail in the reference by Ji and others [13]. The mTPI design is a Bayesian dose finding design that uses the posterior probability in guiding dose selection. The mTPI design uses a statistic for the decision rules called the unit probability mass (UPM), defined as the ratio of the probability mass of the interval and the length of the interval [13]. The toxicity probability scale is divided into three portions: (0, p_T_-ε_1_) corresponding to under-dosing, [p_T_-ε_1_, p_T_+ε_2_] corresponding to proper dosing and (p_T_+ε_2_, 1) corresponding to over-dosing. Here p_T_ is the target probability of dose limiting toxicity and ε_1_ and ε_2_ are used to define the interval for the target DLT rate. The rules for escalating, staying at the same dose or de-escalating depend on which of these portions has the highest UPM for that dose level, based on a beta-binomial distribution with a beta(1,1) prior [13], [14]. For example, the next cohort of patients will be treated at the next higher dose level if the UPM is the largest for the under-dosing interval. The trial stops if dose level 1 is too toxic or if the maximum sample size is reached or exceeded.

The TEQR design is a frequentist version of the mTPI design and is described in detail in the reference by Blanchard and Longmate [14]. This design is not based on the posterior probability but on the empirical DLT rate. The unit interval is divided into three portions: (0, p_T_-ε_1_), [p_T_-ε_1_, p_T_+ε_2_] and (p_T_+ε_2_,1). The rules for escalating, staying at the same dose or de-escalating depend on which of these portions contains the empirical DLT rate for that dose level – if the empirical DLT rate lies between 0 and p_T_-ε_1_, we escalate; if it lies in the interval [p_T_-ε_1_, p_T_+ε_2_], we stay at the same dose; if it lies above p_T_+ε_2_, we de-escalate. In both the mTPI and TEQR design, we stay at the current dose if the current dose is safe but the next higher dose is too toxic based on the data. A trial using the TEQR design stops if dose level 1 is too toxic or when a dose level achieves the selected MTD sample size. In a trial using the TEQR or the mTPI design, the MTD is determined to be the highest dose level with a DLT rate that is closest to (and below) the target DLT rate after applying isotonic regression at the end of the trial.

The concept of the BOIN design is similar to that of the TEQR design in terms of dividing the toxicity probability scale into three intervals and using these intervals along with the empirical DLT rate to guide dose finding [15]. In contrast to the TEQR and mTPI designs, where the interval for the target DLT rate is fixed and is independent of the dose level and the number of patients that have been treated at that dose level, the BOIN design is more general and permits this interval to vary with the dose level and the number of patients that have been treated at that dose level. In this design, the probability of patients being assigned to very toxic doses or to sub-therapeutic doses is low. A trial using the BOIN design usually stops at the pre-planned sample size but the design allows the incorporation of early stopping rules.

The CRM design and its variations are well-known and are described in several references [25], [26], [27], [28]. This design uses the DLT information obtained from all the previous patients to determine the dose level to which the next patient (or cohort of patients [28]) is assigned. The first patient may be given a dose whose DLT rate is expected to be close to the target DLT rate based on information from previous studies, although caution usually dictates starting at a lower dose level. The dose given to each subsequent patient is decided by the DLT data of all the previous patients in conjunction with a dose-toxicity model for e.g. a one parameter logistic model with parameter “a”. The estimates of “a” in the dose-toxicity model are updated using Bayesian methods after the DLT information from each patient is obtained. For example, after n patients are enrolled, $\hat{a_{n}}=\;\int_{0}^{\infty }a\;f\left(a\text{|}{Ω}_{n}\right)da,$where $f\left(a\text{|}{Ω}_{n}\right)=L_{{Ω}_{n}}\left(a\right)g\left(a\right)/\int_{0}^{\infty }L_{{Ω}_{n}}\left(a\right)g\left(a\right)da$;

$f\left(a\text{|}{Ω}_{n}\right)$ is the posterior density of a, g(a) is the prior distribution for a, $L_{{Ω}_{n}}\left(a\right)$ is the likelihood function, and ${Ω}_{n}$ are the DLT data after n patients [29]. The dose-toxicity model is then used to recommend the dose level for the next patient, typically the dose with a DLT rate closest to but less than the updated DLT estimate from the model, subject to not skipping over untested doses. The stopping point for this process is usually the pre-determined sample size of the trial or an observation of no change in dose assignment for a sequence of n patients.

The EWOC design is a Bayesian adaptive dose finding design, whose unique feature is over-dose control i.e. the posterior probability of treating patients at doses above the MTD, given the data, cannot be greater than a certain pre-specified probability α [16], [17]. In mathematical terms, we specify a prior distribution for (ρ_0_, γ), where ρ_0_ is probability of DLT at the minimum dose and γ is the MTD dose, and let Π_n_(γ) be the marginal posterior cdf of γ given D_n_ (DLT data after n patients). The first patient receives the dose x_1_, and conditional on the event of no DLT at x_1_, the (n+1)^th^ patient receives the dose x_n+1_ = Π^−1^_n_(α), which implies that the posterior probability of exceeding the MTD is equal to α [17]. The design also minimizes the under-dosing of patients. This means that the MTD is generally reached rapidly, and after the initial cohorts of patients, the remaining cohorts of patients are treated at dose levels reasonably close to the MTD. In this design, it is also possible to add a stopping rule for excessive toxicity for e.g. the trial will be stopped early if three consecutive DLTs are observed or if the posterior probability at the minimum dose exceeds a certain pre-defined value.

### 2.3 Simulations of rule based designs

For our simulations in SAS of the 3 + 3 design and its extensions, we use a Bernoulli random generator, along with the probability of a DLT at different doses generated by a dose-toxicity curve, to randomly assign each patient a DLT or not depending on the probability of a DLT at the assigned dose. We then implement the assignment rules of each design and follow each simulated trial to its conclusion. For example, for the designs that allow only escalation, we escalate until the number of DLTs at the last dose level examined exceeds that allowed by the specific design, and the MTD is then estimated to be one dose level below the last dose level examined. We perform these simulations 10000 times for each combination of design and dose-toxicity curve. The increase in dose at a new dose level beyond dose level 1 for each dose-toxicity curve investigated is based on the modified Fibonacci series (2, 1.67, 1.5, 1.4, 1.33, 1.33, 1.33 etc.), as commonly used in many oncology trials [25].

A logistic dose-toxicity curve is often used to describe the underlying relation between dose and toxicity in cytotoxic agents [22]. Hence, we specify the true DLT probability at each dose based on a specific logistic curve. In addition to the logistic curve, we consider a specific log logistic and a linear dose-toxicity curve to study the performance sensitivity of these designs to the true DLT probabilities generated by these different dose-toxicity curves. Table 2 shows the true DLT rates at each dose level for each of the three dose-toxicity curves. For determining the two unknown coefficients of each dose-toxicity curve, we use the DLT rates at two different doses – namely we assume a true DLT rate of 0.01 at dose level 1 of 100 units and a DLT rate of 0.2 at the true MTD (dose level 3) of 334 units. We assume a DLT rate of 0.2 at the MTD because the 3 + 3 design targets a DLT rate between 0.2 and 0.25 [19]. Hence this choice of 0.2 allows a fair comparison of the simulation results from the 3 + 3 design with those from other A + B designs whose approximate target DLT rate is 0.2 (various A + B designs target DLT rates other than 0.2; see Table 4.1 of Chapter 4 in the reference by Ting [30]). However, we also study the performance of these designs to different target DLT rates, such as 0.1 and 0.33.

**Table 2:** DLT rates at different doses for the three dose-toxicity curves.

Dose level	Dose	Linear dose-toxicity DLT rate = min(−0.071197 + 0.000811966*dose,1)	Logistic dose-toxicity Log_e_(DLT rate/(1−DLT rate)) = −5.96641 + 0.013713*dose	Log-logistic dose-toxicity Log_e_(DLT rate/(1−DLT rate)) = −16.8485 + 2.66078*log_e_(dose)
		DLT rate	DLT rate	DLT rate
−3	12.5 units		0.00303	0.00004
−2	25		0.0036	0.0003
−1	50		0.00506	0.0016
1	100	0.01	0.01	0.01
2	200	0.09	0.04	0.06
3	334	0.2	0.2	0.2
4	501	0.34	0.71	0.42
5	701.4	0.50	0.97	0.64
6	932.86	0.69	1	0.79
7	1240.71	0.94	1	0.89
8	1650.14	1	1	0.95
9	2194.69	1	1	0.97
10	2918.93	1	1	0.99

We choose the following broad range of statistical operating characteristics to compare and evaluate the dose finding schemes considered for these three dose-toxicity curves: the accuracy of MTD selection, the average number of dose levels examined and its standard deviation, the maximum and median number of dose levels examined, the mean and median number of patients and the median number of DLTs per trial, the mean number of patients dosed at the MTD, the mean percentage of patients dosed at the MTD, above the MTD and below the MTD, the average number of patients and DLTs at each dose level, the average trial DLT rate and the average DLT rate at the MTD. Further, we investigate the effect of the location of the starting dose relative to the true MTD on the accuracy of MTD selection for the chosen logistic and log-logistic dose-toxicity curves for e.g. when we start our trial simulation at dose level −3, −2 or −1 instead of at dose level 1 (see Table 2; these low doses double each time). In addition, we use three linear dose-toxicity curves with different offsets to investigate the effect of the location of the starting dose relative to the true MTD on the accuracy of MTD selection for the 3 + 3 design. Our SAS programs, available on request, are presently able to provide results for six designs (3 + 3, 2 + 4, 4 + 4 a, 5 + 5 a, 3 + 3+3, and simple accelerated titration designs) and three dose-toxicity curves (linear, logistic, log-logistic). However, the programs are simple and flexible and can be extended to other A + B designs as well as any other dose-toxicity curve.

### 2.4 Simulations of model based designs or designs that allow specification of the target DLT rate

We use R code provided by Ji et al. [13] to implement the mTPI design. The program requires the following inputs: number of simulations, target probability of dose limiting toxicity p_T_ and ε_1_ and ε_2_ that help define the lower and upper bound of the interval for the target DLT rate respectively, sample size, cohort size, starting dose and the true DLT rate at each dose.

We use the R package TEQR to implement the TEQR design. The program requires the following inputs: number of simulations, target probability of dose limiting toxicity p_T_ and ε_1_ and ε_2_ that help define the lower and upper bound of the interval for the target DLT rate respectively, DLT probability deemed to be too toxic, desired sample size at the MTD, cohort size, maximum number of cohorts, starting dose and the true DLT rate at each dose.

We use the R package BOIN to implement the BOIN design. The program requires the following inputs: number of simulations, target probability of dose limiting toxicity p_T_, cohort size, number of cohorts, starting dose, cut off to eliminate an overly toxic dose for safety and the true DLT rate at each dose. Although the design allows the possibility of rules for stopping prior to reaching the planned sample size, we did not implement these early stopping rules, to permit fair comparisons between designs.

We use a CRM trial simulator to implement the various scenarios for the CRM design. The program requires the following inputs: number of simulations, maximum sample size, cohort size, number of doses, starting dose, target probability of dose limiting toxicity, stopping probability (the trial is stopped if the probability that the lowest dose is more toxic than the target is greater than this value) and the true DLT rates at the various doses. The probability of DLT at dose i is modeled as p_i_^exp(α)^, where p_i_ is a constant and α is distributed a priori as a normal random variable with mean 0 and variance 2. The initial default prior probabilities of DLT used in the software are given in Appendix Table 3. The trial stops when the planned sample size is reached or if the lowest dose is too toxic.

We use a web based program to implement the EWOC design. The program requires the following inputs: number of simulations, target probability of dose limiting toxicity, maximum acceptable probability of exceeding the target dose (α), variable α increment, cohort size, sample size, minimum dose, maximum dose, number of dose levels and the true probability of DLT at each dose. Although the EWOC design allows the possibility of rules for stopping prior to reaching the planned sample size, the current implementation of the EWOC design does not include early stopping rules.

The parameters used for mTPI, TEQR, BOIN, CRM and EWOC designs are shown in Appendix Tables 2, 3, 4 and 5. Note that the sample size is an output of the rule-based A + B designs as well as the TEQR design. For the mTPI, BOIN, CRM and EWOC designs, we use the same sample size that the TEQR design yields for each of the three sets of true DLT rates.

## 3 Results

### 3.1 Comparison of operating characteristics for designs that target a DLT rate of 0.2

For all the simulation results in this section, dose level 1 is the lowest dose (see Table 2) and dose level 3 is the true MTD.

**Fig. 2:**
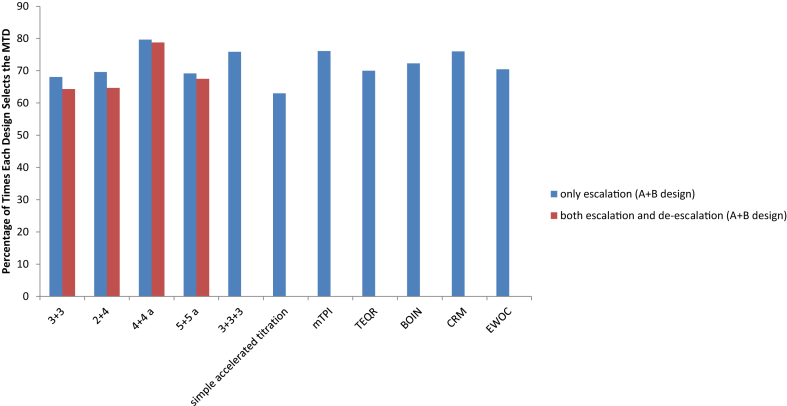
Depicts the percentage of times each design considered selects the MTD (Dose Level 3) for the true DLT rates generated from the logistic dose-toxicity curve given in Table 2. These percentages are from simulations and the results are shown in Table 3, Table 4, Table 5.

For the logistic dose-toxicity curve constructed, there is a very clear separation between the true DLT rate at the MTD and the rates at the dose levels below and above it: the DLT rate of 0.2 at the MTD versus 0.04 at the dose level below and 0.71 at the dose level above (Table 2). The DLT rate of 0.2 at dose level 3 aligns with the range of toxicity rates that the escalation-only A + B designs target (Table 1) and is the target DLT rate specified for the model-based designs. Hence all the designs pick dose level 3 as the MTD the largest percentage of times in our simulations, while incorrectly picking the other dose levels substantially less frequently (Table 3; also see Appendix Table 1 for exact analytic results for MTD selection for the 3 + 3 design and its extensions). The 4+4a design with and without de-escalation, the mTPI design, the CRM design and the 3 + 3+3 design correctly pick dose level 3 as the MTD ∼79%, ∼80%, ∼76%, ∼76% and ∼76% percent of the time respectively (Table 3 and Fig. 2). The median number of patients enrolled in the trial ranges from 6 for the simple accelerated titration design to 25 for the 5 + 5 a design. As expected, with the 3 + 3 design, about half of the patients are given doses below the MTD. The BOIN design and the 5 + 5 a design with and without de-escalation also treat a large percentage of patients at doses below the MTD – about 50%, 48% and 49% respectively. On the other hand, the simple accelerated titration design over-doses a large percentage of patients (∼43%). The model based designs generally treat a large percentage of patients at the MTD. The average trial DLT rate ranges from 0.17 for the TEQR design to 0.4 for the simple accelerated titration design; the median number of DLTs per trial ranges from 2 for the 2 + 4 design without de-escalation to 5 for the 4+4a design with de-escalation and the 5 + 5 a design, among the extensions of the 3 + 3 design considered.

**Table 3:** Simulation results: logistic dose-toxicity: Log_e_(DLT rate/(1−DLT rate)) = −5.96641 + 0.013713*dose.

Design	% of times that dose level 3 is selected as the MTD	% of times that doses below the MTD (dose levels 1 and 2) are selected as the MTD	% of times that doses above the MTD (dose levels 4 and above) are selected as the MTD	Average number of dose levels examined	Std of dose levels examined	Max dose levels examined	Median dose levels examined	Average number of patients per trial	Median number of patients per trial	Median number of dlts per trial	Average sample size at MTD	Average % of pts dosed at MTD	Average % of pts under- dosed	Average % of pts over- dosed
3 + 3^a^	68.05 (64.32)	29.78 (34.75)	2 (0.76)	3.7	0.54	5	4	13.06 (15.53)	12 (15)	3 (3)	4.1 (5.6)	31.43 (35.87)	**50.30 (48.47)**	18.26 (15.66)
2 + 4^a^	69.62 (64.67)	23.54 (34.47)	6.77 (0.77)	3.8	0.56	5	4	10.48 (14.59)	10 (14)	2 (3)	3.22 (5.75)	30.86 (40.17)	43.23 (39.69)	25.90 (20.14)
4 + 4 a^a^	**79.65 (78.79)**	19.39 (20.45)	0.96 (0.75)	3.8	0.42	5	4	19.23 (21.63)	20 (20)	4 (5)	6.24 (7.86)	32.67 (36.68)	46.08 (44.13)	21.26 (19.19)
5 + 5 a^a^	69.19 (67.5)	30.68 (32.43)	0.13 (0.05)	3.7	0.47	5	4	23.14 **(26.12)**	**25 (25)**	5 (5)	8.05 (9.67)	34.96 (37.13)	**48.83 (48.26)**	16.21 (14.61)
3 + 3+3	**75.9**	21.77	2.3	3.8	0.47	5	4	13.96	15	3	4.59	32.25	47.72	20.03
Simple accelerated titration	62.98	14.51	22.43	4.1	0.64	6	4	**7.14**	**6**	3	1.88	24.90	32.06	**43.04**
mTPI	**76.1**	23	0.85	5		5	5	21 (max)	21 (max)		10.1	**47.88**	41.9	10.22
TEQR	70	27	1	5		5	5	21.78	21		9.5	**43.66**	46.74	9.6
BOIN	72.3	25.4	2.3	5		5	5	21 (max)	21 (max)	3.4 (mean)	8.6	41.15	**49.76**	9.09
CRM	**76**	21	3	5		5	5	21 (max)	21 (max)	3.6 (mean)	9.8	**46.88**	43.97	9.15
EWOC	70.45	9.7	19.85	5		5	5	21 (max)	21 (max)		10.1	**48.04**	40.06	11.9

The bold highlighting shows the designs predicted by simulations to pick the MTD most accurately, to enroll the largest and smallest number of patients, to dose the maximum percentage of patients at the MTD, to under-dose the maximum percentage of patients, and to over-dose the maximum percentage of patients. Note also that the sum of columns 2 to 4 may add up to <100% because the remaining small percentage of times, no dose level is selected as the MTD.

a The numbers shown in brackets are for a corresponding design that also allows dose de-escalation.

For the log-logistic dose-toxicity curve constructed, there is a clear separation between the true DLT rate at the MTD and the rates at the dose levels below and above it: the DLT rate of 0.2 at the MTD versus 0.06 at the dose level below and 0.42 at the dose level above (Table 2). Although this separation is not as large as it is in the logistic dose-toxicity curve considered, all the designs still pick dose level 3 as the MTD more frequently than they pick any other dose level. The CRM, mTPI, BOIN and 5 + 5 a with and without de-escalation designs correctly pick dose level 3 as the MTD ∼74%, ∼63%, ∼59%, ∼58% and ∼58% percent of the time respectively (Table 4). The median number of patients enrolled in the trial ranges from 7 for the simple accelerated titration design to 30 for the 5 + 5 a design with de-escalation. For this dose-toxicity curve, about 49% of patients are given doses below the MTD in the 3 + 3 design. The BOIN, TEQR and 5 + 5 a design with and without de-escalation also treat a large percentage of patients at doses below the MTD – about 50%, 47%, 47% and 47% respectively. On the other hand, the simple accelerated titration design over-doses a large percentage of patients (∼47%). The model based designs generally treat a large percentage of patients at the MTD. The average trial DLT rate ranges from 0.17 for the TEQR design to 0.34 for the simple accelerated titration design; the median number of DLTs per trial ranges from 2 for the simple accelerated titration design, reflecting the very small sample size for this design, to 5 for the 4 + 4 a design and the 5 + 5 a design with de-escalation, among the extensions of the 3 + 3 design considered.

**Table 4:** Simulation results: log-logistic dose-toxicity: Log_e_(DLT rate/(1−DLT rate)) = −16.8485 + 2.66078*log_e_(dose).

Design	% of times that dose level 3 is selected as the MTD	% of times that doses below the MTD (dose levels 1 and 2) are selected as the MTD	% of times that doses above the MTD (dose levels 4 and above) are selected as the MTD	Average number of dose levels examined	Std of dose levels examined	Max dose levels examined	Median dose levels examined	Average number of patients per trial	Median number of patients per trial	Median number of DLTs per trial	Average sample size at MTD	Average % of pts dosed at MTD	Average % of pts under- dosed	Average % of pts over- dosed
3 + 3^a^	49.45 (50.55)	31.66 (35.95)	18.72 (13.38)	3.8	0.8	7	4	14.2 (16.73)	15 (15)	3 (3)	4.00 (5.18)	28.72 (31.16)	**48.61 (47.44)**	22.67 (21.4)
2 + 4^a^	45.8 (50.89)	24.48 (33.94)	29.6 (15.05)	4.1	0.87	8	4	11.89 (16.29)	12 (16)	3 (3)	3.16 (5.19)	27.49 (32.71)	40.05 (37.8)	32.46 (29.49)
4 + 4 a^a^	56.73 (57.76)	20.26 (20.69)	23.01 (21.54)	4	0.7	6	4	21.96 (24.23)	20 (24)	5 (5)	6.18 (7.4)	29.09 (31.3)	42.78 (41.49)	28.13 (27.21)
5 + 5 a^a^	**58.07 (58.09)**	31.38 (33.18)	10.54 (8.71)	3.8	0.65	6	4	25.54 **(28.43)**	25 **(30)**	4 (5)	7.96 (9.37)	31.95 (33.38)	**46.85 (46.63)**	21.21 (19.99)
3 + 3+3	53.96	22.43	23.56	4	0.74	7	4	15.89	15	3	4.55	28.9	44.54	26.56
Simple accelerated titration	36.32	15.67	47.95	4.5	1.05	9	4	**8.11**	**7**	2	1.87	22.93	29.81	**47.25**
mTPI	**63.15**	22.45	14.35	7		7	7	24 (max)	24 (max)		10.0	**41.67**	40.49	17.85
TEQR	57	32	8	7		7	7	22.71	24		8.6	**37.78**	**46.98**	15.24
BOIN	**59.2**	28	12.7	7		7	7	24 (max)	24 (max)	3.7 (mean)	8.9	**37.08**	**50**	12.92
CRM	**74**	18	8	7		7	7	24 (max)	24 (max)	4.0 (mean)	10.1	**41.92**	43.42	14.67
EWOC	57.1	9.7	33.2	7		7	7	24 (max)	24 (max)		11.4	**47.32**	22.92	29.76

The bold highlighting shows the designs predicted by simulations to pick the MTD most accurately, to enroll the largest and smallest number of patients, to dose the maximum percentage of patients at the MTD, to under-dose the maximum percentage of patients, and to over-dose the maximum percentage of patients. Note also that the sum of columns 2 to 4 may add up to <100% because the remaining small percentage of times, no dose level is selected as the MTD.

a The numbers shown in brackets are for a corresponding design that also allows dose de-escalation.

For the linear dose-toxicity curve constructed, the DLT rate at dose level 3 is 0.2 and the DLT rate at dose level 4 is 0.34 (Table 2). Although this separation is even smaller than that in the logistic and log-logistic dose-toxicity curves considered, all the designs except the accelerated titration design (which picks dose level 3 as the MTD 27% of the time versus dose level 4 as the MTD 29% of the time) pick dose level 3 as the MTD more frequently than any other dose level. The CRM, mTPI, 5 + 5 a with and without de-escalation and TEQR designs correctly pick dose level 3 as the MTD but only ∼54%, ∼45%, ∼45%, ∼45% and ∼45% percent of the time respectively (Table 5). The median number of patients enrolled in the trial ranges from 8 for the simple accelerated titration design to 30 for the 5 + 5 a design with de-escalation. For this dose-toxicity curve, about half of the patients are given doses below the MTD in the 3 + 3 design. The BOIN, TEQR, CRM, mTPI designs and the 5 + 5 a design with and without de-escalation also treat a large percentage of patients at doses below the MTD – about 58%, 50%, 50%, 48%, 48% and 48% respectively. On the other hand, the simple accelerated titration over-doses a large percentage of patients (∼49%). The model based designs generally treat a large percentage of patients at the MTD. The average trial DLT rate ranges from 0.16 for the TEQR design to 0.31 for the simple accelerated titration design; the median number of DLTs per trial ranges from 2 for the simple accelerated titration design to 5 for the 4 + 4 a and 5 + 5 a designs, among the extensions of the 3 + 3 design.

**Table 5:** Simulation results: linear dose-toxicity: DLT rate = min(−0.071197 + 0.000811966^∗^dose, 1).

Design	% of times that dose level 3 is selected as the MTD	% of times that doses below the MTD (dose levels 1 and 2) are selected as the MTD	% of times that doses above the MTD (dose levels 4 and above) are selected as the MTD	Average number of dose levels examined	Std of dose levels examined	Max dose levels examined	Median dose levels examined	Average number of patients per trial	Median number of patients per trial	Median number of DLTs per trial	Average sample size at MTD	Average % of pts dosed at MTD	Average % of pts under- dosed	Average % of pts over- dosed
3 + 3^a^	37.49 (39.86)	34.6 (37.62)	27.72 (22.39)	3.9	1.01	7	4	14.75 (17.22)	15 (18)	3 (3)	3.85 (4.76)	26.44 (27.73)	**49.64 (48.67)**	23.92 (23.60)
2 + 4^a^	34.59 (39.72)	26.88 (33.93)	38.42 (26.27)	4.2	1.1	7	4	12.52 (16.9)	12 (16)	3 (3)	3.08 (4.63)	25.52 (28.2)	40.75 (38.7)	33.73 (33.1)
4 + 4 a^a^	40.56 (41.94)	21.47 (21.68)	37.97 (36.36)	4.2	0.92	7	4	23.64 (25.78)	24 (24)	5 (5)	6.07 (6.97)	26.73 (27.96)	42.52 (41.28)	30.75 (30.76)
5 + 5 a^a^	**44.59 (45.44)**	33.92 (35.13)	21.48 (19.41)	3.8	0.85	6	4	26.85 **(29.63)**	25 **(30)**	5 (5)	7.66 (8.74)	29.24 (29.88)	**47.87 (47.9)**	22.89 (22.23)
3 + 3+3	39.56	24.73	35.63	4.1	0.97	7	4	16.99	18	3	4.43	26.57	44.55	28.89
Simple accelerated titration	26.69	16.99	56.26	4.7	1.26	8	5	**8.67**	**8**	2	1.85	21.5	29.94	**48.57**
mTPI	**45.3**	28.6	26.05	7		7	7	21 (max)	21 (max)		6.9	**32.71**	**47.99**	19.29
TEQR	**45**	37	15	7		7	7	22.88	21		7.4	**32.12**	**49.78**	18.09
BOIN	40.4	38.1	21.6	7		7	7	21 (max)	21 (max)	3.0 (mean)	6.1	29.05	**57.62**	13.33
CRM	**54**	24	22	7		7	7	21 (max)	21 (max)	3.3 (mean)	7.2	**34.43**	**49.57**	16.00
EWOC	40.35	8.90	50.75	7		7	7	21 (max)	21 (max)		8.5	**40.39**	23.81	35.81

The bold highlighting shows the designs predicted by simulations to pick the MTD most accurately, to enroll the largest and smallest number of patients, to dose the maximum percentage of patients at the MTD, to under-dose the maximum percentage of patients, and to over-dose the maximum percentage of patients. Note also that the sum of columns 2 to 4 may add up to <100% because the remaining small percentage of times, no dose level is selected as the MTD.

a The numbers shown in brackets are for a corresponding design that also allows dose de-escalation.

**Fig. 3:**
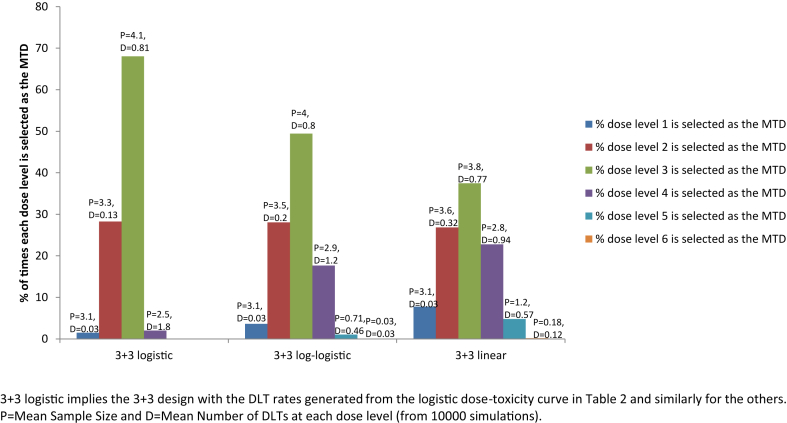
Depicts the percentage of times that the 3 + 3 design selects each dose level as the MTD for the true DLT rates given in Table 2, generated from the three dose-toxicity curves. These percentages are from simulations and the results are shown in Table 3, Table 4, Table 5.

**Appendix Fig. 1:**
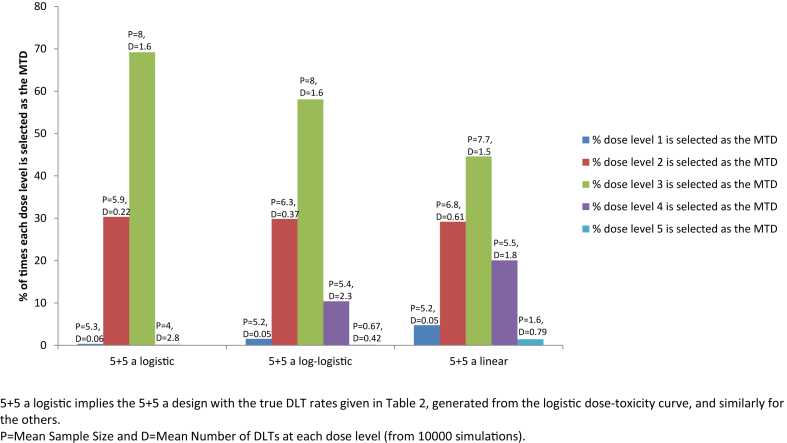
The figure depicts the percentage of times that the 5+5 a design selects each dose level as the MTD for the true DLT rates given in Table 2, generated from the three dose-toxicity curves. These percentages are from simulations and the results are shown in Table 3, Table 4, Table 5.

**Appendix Fig. 2:**
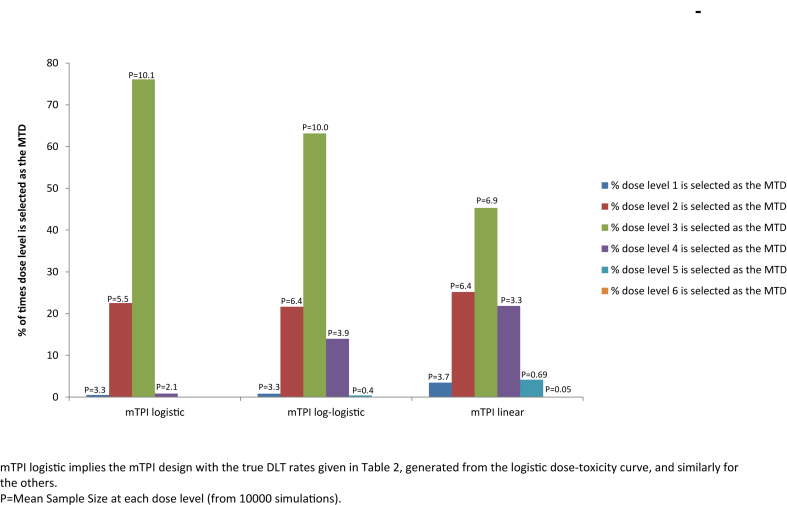
The depicts the percentage of times that the mTPI design selects each dose level as the MTD for the true DLT rates given in Table 2, generated from the three dose-toxicity curves. These percentages are from simulations and the results are shown in Table 3, Table 4, Table 5.

**Appendix Fig. 3:**
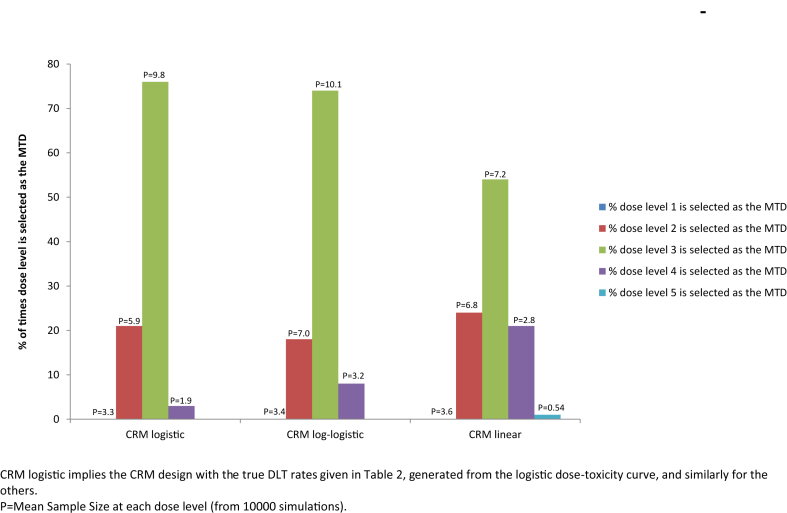
The figure depicts the percentage of times that the CRM design selects each dose level as the MTD for the true DLT rates given in Table 2, generated from the three dose-toxicity curves. These percentages are from simulations and the results are shown in Table 3, Table 4, Table 5.

Results for the accuracy of MTD selection for the 3 + 3 design for all the three dose-toxicity curves considered are presented in Fig. 3; results for some of the other designs are presented graphically in Appendix Figs. 1–3.

### 3.2 Effect of starting the trial at lower dose levels on the accuracy of MTD selection

In the previous section, our simulations are started at dose level 1 for all the rule-based designs, and dose level 3 is the true MTD for all the designs. This means that it takes only two escalations from the starting dose to reach the true MTD in the escalation only designs. However, the accuracy of MTD selection could depend on where the starting dose is located relative to the true MTD, for example if it is located six dose levels below the true MTD versus two, because some dose finding designs may be slow to escalate while others may be fast to do so. Thus, we investigate the effect of starting at lower dose levels on the accuracy of MTD selection in the 3 + 3 design and its extensions that allow only escalation, using the logistic dose-toxicity curve in Table 2. We find that the number of patients on the trial and the percentage of patients who are under-dosed, both of which are outputs of the program for the rule-based designs, increase when we start at the lower doses, but the accuracy of MTD selection is largely unaffected for all these designs (Table 6). We find similar results for the model based designs. We also find similar results for the log-logistic dose-toxicity curve in Table 2 to those described for the logistic dose-toxicity curve. The result that the location of the starting dose relative to the true MTD does not affect the accuracy of MTD selection may not be surprising since the true DLT rates at dose level −1, −2 and −3 are very small for the logistic and log-logistic dose-toxicity curves used.

**Table 6:** Simulation results: logistic dose-toxicity: Log_e_(DLT rate/(1−DLT rate)) = −5.96641 + 0.013713*dose: effect of starting at lower doses on the accuracy of MTD selection.

Design	Median sample size when starting dose is dose level −3	Accuracy of MTD selection when starting dose is dose level −3 (% of times dose level 3 is selected as MTD)	% of patients underdosed when starting dose is dose level −3	Median sample size when starting dose is dose level −2	Accuracy of MTD selection when starting dose is dose level −2 (% of times dose level 3 is selected as MTD)	% of patients underdosed when starting dose is dose level −2	Median sample size when starting dose is dose level −1	Accuracy of MTD Selection when starting dose is dose level −1 (% of times dose level 3 is selected as MTD)	% of Patients underdosed when starting dose is dose level −1	Median sample size when starting dose is dose level 1	Accuracy of MTD Selection when starting dose is dose level 1 (% of times dose level 3 is selected as MTD)	% of patients underdosed when starting dose is dose level 1
3 + 3	21	67.79%	70.84%	18	67.11%	66.16%	15	67.82%	59.74%	12	68.05%	50.30%
2 + 4	16	70.45%	63.96%	14	70.51%	59.06%	12	69.76%	52.45%	10	69.62%	43.23%
4 + 4 a	32	79.59%	67.02%	28	79.54%	62.21%	24	79.53%	55.66%	20	79.65%	46.08%
5 + 5 a	40	69.48%	69.18%	35	69.05%	64.48%	30	69.80%	58.22%	25	69.19%	48.83%
3 + 3+3	24	75.92%	68.45%	21	75.85%	63.66%	18	76.09%	57.14%	15	75.9%	47.72%
Accelerated titration	9	63.35%	52.96%	8	63.79%	47.60%	7	63.00%	41.04%	6	62.98%	32.06%
mTPI	30 (max)	77.3%	59.71%	27 (max)	77.5%	54.49%	24 (max)	77.8%	48.72%	21 (max)	76.1%	41.9%
TEQR	30	70%	62.91%	27	69%	59.17%	24	71%	54.22%	21	70%	46.74%
BOIN	30 (max)	72.2%	65.89%	27 (max)	71%	62.08%	24 (max)	72%	57.5%	21 (max)	72.3%	49.76%
CRM	30 (max)	75%	60.88%	27 (max)	76%	56.2%	24 (max)	76%	50.73%	21 (max)	76%	43.97%
EWOC	30 (max)	70.2%	52.76%	27 (max)	70.85%	45.76%	24 (max)	65.3%	47.4%	21 (max)	70.45%	40.06%

The sample size is an output for the A + B escalation only designs. For the model based designs, the sample size is an output for the TEQR design and we use the same sample size obtained from the TEQR design for the other model based designs. For the CRM design, a prior DLT rate of 0.01, 0.05 and 0.1 are used at dose levels −1, −2 and −3.

**Appendix Fig. 4:**
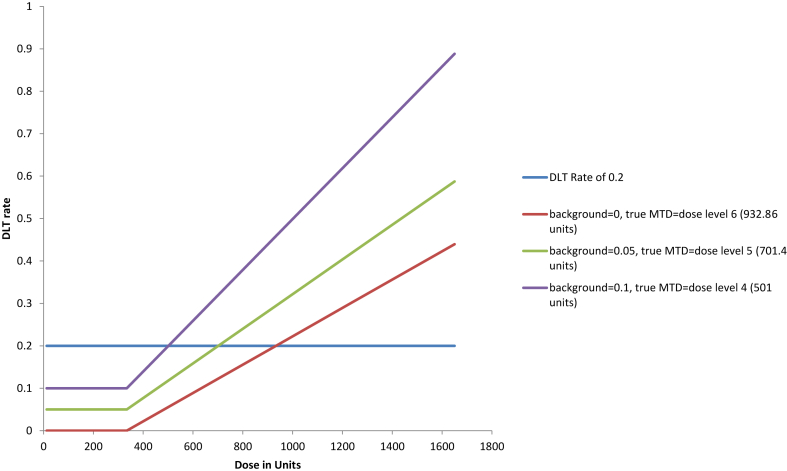
The figure depicts three linear dose-toxicity curves with different offsets that are used to investigate the effect of the location of the starting dose relative to the true MTD on the accuracy of MTD selection for the 3+3 design.

In general, the accuracy of MTD selection will be affected when the true DLT rates at these lower dose levels are much greater than 0.01 (say 0.1). We have demonstrated this for the 3 + 3 design using three linear dose-toxicity curves with different offsets (see Appendix Table 8 and Appendix Fig. 4). In practice, the starting dose of the trial is usually an extremely conservative estimate based on animal studies, and the DLT rates at the first few dose levels are expected to be very low.^1^ In this case, the accuracy of MTD selection should not be affected even when the true MTD is several doses above the starting dose in the rule-based escalation only designs considered, and we can enroll patients at the same low starting dose for these designs.

## 4 Discussion

In this work, we have systematically compared via simulations the statistical operating characteristics of various Phase I oncology designs, namely the 3 + 3 design and its extensions that target a DLT rate of ∼0.2 as well as the mTPI, TEQR, BOIN, CRM and EWOC designs with a pre-specified target DLT rate of 0.2, for three sets of true DLT rates (generated for the same doses from a specific linear, logistic and log-logistic dose-toxicity curve). Although this is not an exhaustive comparison of all the current Phase 1 oncology designs, we have covered multiple commonly used ones. The 3 + 3 design is very simple and easy to implement and hence is still commonly used. However, our simulations show, not unexpectedly, that it under-doses a large percentage of patients, and is also not the design that picks the MTD most accurately for any of the dose-toxicity curves examined, with or without de-escalation.

All the designs examined select the MTD fairly accurately when there is a clear separation between the true DLT rate at the MTD and the rates at the dose level immediately below and above it, as is the case for the DLT rates generated using the chosen logistic dose-toxicity curve. However, when this separation is small, as is the case for the DLT rates generated using the chosen linear dose-toxicity curve, the accuracy of MTD selection is much lower. The separations in these true DLT rates depend, in turn, not only on the functional form of the dose-toxicity curve but also on the investigated dose levels and the parameter set-up. The considered A + B designs with de-escalation generally pick the MTD more accurately than the corresponding escalation-only design for the true DLT rates generated using the chosen log-logistic and linear toxicity curves, but not for the logistic one. Some of the other rule based designs examined pick the MTD more accurately than the 3 + 3 design, depending on the true DLT rate at each dose. For example, the 5 + 5 a design is as accurate as the model based designs in picking the MTD for the true DLT rates generated using the chosen log logistic and linear dose-toxicity curves but requires enrolling a larger number of patients compared to the other designs considered (∼30 patients) and under-doses a large percentage of patients (∼48%) for these dose-toxicity curves. Among the designs investigated, the simple accelerated titration design over-doses a large percentage of patients. Over-dosing of patients in oncology trials is an important issue that needs to be considered carefully in terms of study design since the toxicities at the higher doses can be very harmful to patients. The EWOC design explicitly takes this into consideration; in this design, one can control the expected proportion of patients receiving doses above the MTD by pre-specifying the maximum acceptable probability of exceeding the target dose. Although some model-based designs can be more difficult to implement than rule based designs, the model based designs studied, mTPI, TEQR, BOIN, CRM and EWOC designs, perform well and assign the maximum percentage of patients to the MTD, and also have a reasonably high probability (given the small sample size) of picking the true MTD.

In our simulations, we assumed a true DLT rate of 0.2 at the MTD (dose level 3) because it has been shown that the standard 3 + 3 design targets a toxicity rate between 0.2 and 0.25 [19]. However, when a DLT rate of 0.1 is specified as the target DLT rate, the various A + B designs considered would not, in general, select the MTD accurately because 0.1 is not within their target range, and when a DLT rate of 0.33 or 0.4 at the MTD is assumed, A + B designs that target a higher DLT rate would pick the MTD correctly more often than the 3 + 3 design. For example, for the linear dose-toxicity curve in Table 2, dose level 2 is the true MTD if the target DLT rate is 0.1. In this case and for the extensions of the 3 + 3 design considered, percentages for correct MTD identification for dose level 2 are lower than those for dose level 3 and range from 14% (accelerated titration design) to 29% (5 + 5 a with target range 0.2–0.25); percentage for 3 + 3 is 27% (target range 0.17–0.26). If we consider a 5 + 5 design that targets a DLT range of 0.1–0.15 (see Table 4.1 of Chapter 4 of the reference by Ting [30]), it selects dose level 2 as the MTD ∼43% of the time, which is much higher than the percentages with which the 3 + 3 and the other A + B designs with a target DLT rate of ∼0.2 select dose level 2 as the MTD (results for this 5 + 5 design are not included in any table). Dose level 4 is the true MTD if the target DLT rate is 0.33. If we consider the 4 + 4 b design (target range 0.38–0.44) and 5 + 5 b design (target range 0.3–0.35) (see Table 4.1 of Chapter 4 of the reference by Ting [30]), they both select dose level 4 as the MTD ∼40% of the time (results not shown here). This is much higher than the percentages with which the 3 + 3 and the other A + B designs with a target DLT rate of ∼0.2 select dose level 4 as the MTD for the chosen linear dose-toxicity curve (percentages for correct MTD identification range from 20% to 31%). Results for the accuracy of MTD selection for the model based designs for the linear dose-toxicity curve given in Table 2 and for the target DLT rates of 0.1 and 0.33 are provided in Appendix Tables 6 and 7 respectively. The accuracy of MTD selection decreases as the target DLT rate increases from 0.1 to 0.33 for the mTPI, TEQR, BOIN and CRM designs, but not for the EWOC design, for the chosen linear dose-toxicity curve. Our simulations for the A + B and model based designs show that for designs where the approximate DLT rate targeted by the design is known, it is critical to pick a design that is aligned with the true DLT rate of interest.

We also showed that as long as the true DLT rates at the first few dose levels are very low, the accuracy of MTD selection is largely unaffected by the number of escalations it takes to reach the true MTD, for the rule-based escalation only designs considered that target a DLT rate of ∼0.2.

For the standard 3 + 3 design, our simulations, where the starting dose is two levels below the true MTD, show that the maximum number of dose levels examined varies between 5 for the logistic dose-toxicity curve and 7 for the linear and log-logistic dose-toxicity curves considered, while the median number of dose levels examined is 4 for all the three dose-toxicity curves. In comparison, a literature review of 41 trials that were performed using the standard 3 + 3 design found that the median number of dose levels examined was 6 (range 2–12 dose levels), about 45% of the patients were under-dosed and about 20% of the patients were over-dosed [31]. These empirical results are consistent with our simulation findings that the 3 + 3 design under-doses about 50% of the patients and over-doses about 22% of the patients on the trial, for all the three dose-toxicity curves. The average number of patients enrolled in trials that are based on the 3 + 3 design is, however, much higher in the literature review with a mean of 44 patients than in our simulations, where we found a mean of ∼14 patients for all the three dose-toxicity curves. However, this literature review is based on trials of targeted anti-cancer agents that reached the MTD and we do not know the exact percentage of trials that included expansion cohorts, and if the initial cohorts started at very low doses; hence, the above comparisons are not exact. Nevertheless, it is clear from clinical trial data as well as our simulations that Phase I trials are very small and thus may not provide good estimates of the MTD. If we consider designs with a higher average sample size, say 50–60 patients, they will have a much higher accuracy of MTD selection. In the future, it may be worthwhile investing in the enrollment of a larger number of patients even in a Phase I trial to obtain more accurate estimates of the right dose to be used for later Phase trials, although there is always a trade-off between costs (lower number of patients) and more accurate estimates (higher number of patients).

### 4.1 Conclusions

In conclusion, our comprehensive study compares and contrasts the 3 + 3 design with multiple other Phase I oncology designs with an approximate target DLT rate of 0.2 for various scenarios of true underlying DLT rates, in order to understand which designs pick the true MTD most accurately, which under-dose and over-dose the maximum percentage of patients, which assign the maximum number and percentage of patients to the MTD cohort, which explore the maximum number of dose levels and enroll the most number of patients in each case. Our SAS programs are flexible and can be extended to include other A + B designs, other dose-toxicity curves as well as other evaluation criteria. The summaries in this paper provide considerable information on design property trade-offs, and the means to explore additional settings. These may be useful aids in choosing a Phase I design for a particular setting.

## Figures and Tables

**Appendix Table 1:** Analytic results for MTD selection.

Dose level	Probability of DLT	Probability of being the highest dose level examined				
		3 + 3	2 + 4	4 + 4 a	5 + 5 a	3 + 3+3
Logistic dose-toxicity curve						
1	0.01	0.00	0.00	0.00	0.00	0.00
2	0.04	0.02	0.01	0.00	0.01	0.01
3	0.2	0.29	0.23	0.19	0.30	0.21
**4**	**0.71**	**0.68**	**0.70**	**0.79**	**0.69**	**0.76**
5	0.97	0.02	0.07	0.01	0.00	0.02
Log-logistic dose-toxicity curve						
1	0.01	0.00	0.00	0.00	0.00	0.00
2	0.06	0.04	0.03	0.01	0.02	0.02
3	0.2	0.28	0.22	0.19	0.30	0.21
**4**	**0.42**	**0.50**	**0.46**	**0.57**	**0.58**	**0.53**
5	0.64	0.17	0.25	0.22	0.10	0.22
6	0.79	0.01	0.04	0.01	0.00	0.01
Linear dose-toxicity curve						
1	0.01	0.00	0.00	0.00	0.00	0.00
2	0.09	0.08	0.06	0.03	0.05	0.04
3	0.2	0.27	0.21	0.19	0.29	0.21
**4**	**0.34**	**0.38**	**0.34**	**0.40**	**0.45**	**0.39**
5	0.5	0.23	0.27	0.32	0.20	0.29
6	0.69	0.05	0.10	0.06	0.02	0.07
7	0.94	0.00	0.01	0.00	0.00	0.00

These are exact analytic results for MTD selection for extensions of the 3 + 3 design that allow only escalation, and the results are very close to those provided in Table 3, Table 4, Table 5 for MTD selection, which are based on simulations. The rows highlighted in bold show the probability of dose level 3 being chosen as the MTD for the various designs and dose-toxicity curves.

**Appendix Table 2:** Parameters for the mTPI and TEQR designs.

Parameter	mTPI design	TEQR design
Number of simulations	2000	2000
Target toxicity probability p_T_	0.2	0.2
ε_1_	0.05	0.05
ε_2_	0.05	0.05
Starting dose	Dose level 1	Dose level 1
Cohort size	3	3
Sample size	Same as the median sample size obtained from TEQR design	Median sample size is automatically determined (not an input)
Number of dose levels	Same as the maximum dose levels examined (obtained from simulations) for the 3 + 3 design	Same as the maximum dose levels examined (obtained from simulations) for the 3 + 3 design
DLT probability deemed to be too toxic to allow further study at that dose level	NA	0.34
Desired sample size at MTD	NA	12
Maximum number of cohorts	NA	30
True DLT rate at each dose level	Values from Table 2 for each dose-toxicity curve	Values from Table 2 for each dose-toxicity curve

The mTPI software (R code) is available at: http://health.bsd.uchicago.edu/yji/software2.htm.

R code for the TEQR design was developed using the package TEQR.

**Appendix Table 3:** Parameters for the BOIN design.

Parameter	BOIN design
Number of simulations	2000
Target toxicity probability p_T_	0.2
The interval for the target toxicity probability	Used the Default Interval Determined by the design, which is (0.16, 0.24) for p_T_ = 0.2, and is very close to the interval (0.15, 0.25) used for the other model based designs.
Starting dose	Dose level 1
Cohort size	3
Sample size	Same as the median sample size obtained from the TEQR design (the sample size is not a direct input of the program but the number of cohorts is an input and we input the number of cohorts such that the number of cohorts*cohort size is the desired sample size).
Number of cohorts	Desired sample size/cohort size
Cut off to eliminate an overly toxic dose for safety	0.95
True DLT rate at each dose level	Values from Table 2 for each dose-toxicity curve

R code for the BOIN design was developed using the package BOIN.

**Appendix Table 4:** Parameters for the CRM design Used in CRMTrialSimulator.

Parameter	CRM design
Number of simulations	2000
Max sample size	Same as the median sample size obtained from the TEQR design
Cohort size	3
Number of dose levels planned	Same as the maximum dose levels examined (obtained from simulations) for the 3 + 3 design
Starting dose	Dose level 1
Target toxicity probability	0.2
True DLT rate at each dose level	Values from Table 2 for each dose-toxicity curve
**CRM Inputs:**	
The probability of toxicity at dose i is modeled as p_i_^exp(α)^, where p_i_ is a constant and α is distributed a priori as a normal random variable	α is normally disturbed with mean 0 and variance 2
Prior probabilities of toxicity used are the defaults in the program	at dose level 1 = 0.15, at dose level 2 = 0.25, at dose level 3 = 0.3, at dose level 4 = 0.45, at dose level 5 = 0.51, at dose level 6 = 0.56, at dose level 7 = 0.6
Stopping probability (the trial is stopped if the probability that the lowest dose is more toxic than the target is greater than this value)	0.9

The software can be found at: https://biostatistics.mdanderson.org/SoftwareDownload/SingleSoftware.aspx?Software_Id=13.

After the first cohort, each successive cohort is given the dose whose posterior probability of toxicity given the data collected thus far is closest to the target, subject to one additional requirement: one cannot skip over an untried dose. If the method would otherwise skip over an untried dose, the lowest untried dose is given instead.

**Appendix Table 5:** Parameters for the EWOC design used in Web-EWOC simulator.

Parameter	EWOC design
Number of simulations	2000
Sample size	Same as the median sample size obtained from the TEQR design
Cohort size	3
Number of dose levels planned	Same as the maximum dose levels examined (obtained from simulations) for the 3 + 3 design
Starting dose	Dose level 1
Target probability of dose limiting toxicity	0.2
Probability of exceeding target dose (α)	0.25
Variable α increment (resource to control the dose escalation rate in the beginning of the trial)	0.04
Minimum dose and Maximum dose	100 and 500 are the default values (the allowable range is 0–500) and the doses are equally spaced
True DLT rate at each dose level	Values from Table 2 for each dose-toxicity curve
Prior distribution	ρ_0_ ∼ Uniform(0, 0.2) (the prior for ρ_0_, the probability of DLT at the minimum dose, is Uniform(0, 0.2)) γ ∼ Uniform(100, 500) (the prior for the maximum tolerated dose γ is Uniform(100, 500))

The EWOC software is available at: https://biostatistics.csmc.edu/ewoc/ewocWeb.php.

**Appendix Table 6:** Simulation results: linear dose-toxicity: DLT rate = min(−0.071197 + 0.000811966*dose, 1) – Target DLT rate = 0.1for the model-based designs and dose level 2 is the True MTD.

Design	% of times that dose level 2 is selected as the MTD	% of times that doses below the MTD (dose level 1) are selected as the MTD	% of times that doses above the MTD (dose levels 3 and above) are selected as the MTD	Average number of dose levels examined	Std of dose levels examined	Max dose levels examined	Median dose levels examined	Average number of patients per trial	Median number of patients per trial	Median number of DLTs per trial	Average sample size at MTD	Average % of pts dosed at MTD	Average % of pts under- dosed	Average % of pts over- dosed
3 + 3	26.85	7.75	65.21	3.9	1.01	7	4	14.75	15	3	3.6	24.75	20.91	54.34
mTPI	55.75	13.7	30.5	7		7	7	24 (max)	24 (max)		9.5	39.4	22.76	37.84
TEQR	50	15	31	7		7	7	22.81	24		8.8	38.58	23.63	37.79
BOIN	55.9	14.5	29.5	7		7	7	24 (max)	24 (max)	2.9 (mean)	9.6	39.83	26.97	33.2
CRM	57	24	20	7		7	7	24 (max)	24 (max)	2.3 (mean)	8.9	37.03	36.24	26.73
EWOC	43.35	4.15	52.5	7		7	7	24 (max)	24 (max)		8.1	33.93	17.87	48.19

The sum of columns 2 to 4 may add up to <100% because the remaining small percentage of times, no dose level is selected as the MTD.

The default interval for the target DLT rate in the R package is used for the BOIN design.

**Appendix Table 7:** Simulation results: linear dose-toxicity: DLT rate = min(−0.071197 + 0.000811966*dose, 1) – Target DLT rate = 0.33 for the model-based designs and dose level 4 is the True MTD.

Design	% of times that dose level 4 is selected as the MTD	% of times that doses below the MTD (dose levels 3 and below) are selected as the MTD	% of times that doses above the MTD (dose levels 5 and above) are selected as the MTD	Average number of dose levels examined	Std of dose levels examined	Max dose levels examined	Median dose levels examined	Average number of patients per trial	Median number of patients per trial	Median number of DLTs per trial	Average sample size at MTD	Average % of pts dosed at MTD	Average % of pts under- dosed	Average % of pts over- dosed
3 + 3	22.77	72.09	4.95	3.9	1.01	7	4	14.75	15	3	2.8	19.14	71.76	9.1
mTPI	44.1	42.2	13.7	7		7	7	24 (max)	24 (max)		6.0	24.98	67.16	7.86
TEQR	31	65	4	7		7	7	24.57	24		6.1	24.75	65.73	9.52
BOIN	43.2	39.5	17.2	7		7	7	24 (max)	24 (max)	5.1 (mean)	6.0	25	65.83	9.17
CRM	53	32	15	7		7	7	24 (max)	24 (max)	5.7 (mean)	7.2	30	59.38	10.63
EWOC	48.15	11.05	40.8	7		7	7	24 (max)	24 (max)		8.0	33.44	43.27	23.29

The sum of columns 2 to 4 may add up to <100% because the remaining small percentage of times, no dose level is selected as the MTD.

The default interval for the target DLT rate in the R package is used for the BOIN design.

**Appendix Table 8:** Effect of the location of the starting dose relative to the true MTD on the accuracy of MTD selection for the 3 + 3 design for the three linear dose-toxicity curves with different offsets shown in Appendix Fig. 4.

Background DLT rate	Starting dose level relative to true MTD				
	−6	−5	−4	−3	−2
0.1	21.27%	23.91%	26.43%	29.24%	32.36%
0.05	26.41%	28.22%	28.05%	29.45%	29.69%
0	29.86%	31.07%	30.44%	30.9%	30.38%

−6 implies that the starting dose is 6 dose levels below the true MTD, and similarly for the others. We observe that for an offset of 0 (when the true DLT rate = 0 for the first 6 dose levels), the accuracy of MTD selection is not affected by how many dose levels below the true MTD the starting dose level is located i.e. the percentage of times (out of 10000 simulations) that dose level 6 (true MTD) is selected as the MTD is constant (∼30%) for the different starting dose locations relative to the true MTD. However for an offset of 0.1 (when the true DLT rate = 0.1 for the first 6 dose levels), the accuracy of MTD selection is affected by how many dose levels below the true MTD the starting dose level is located.

## Footnotes

1 While this is generally true, there are cases where the true DLT rate at low doses may not be close to zero, such as the following: 1) Phase 1 dose-finding trials sometimes consider all causality DLTs 2) The phrase “adverse events possibly related to study drug” in the definition of a DLT is considered to be “adverse events related to study drug”, and it is often difficult to conclude whether an adverse event is due to the disease or the study drug. 3) The Phase 1 trial escalates a new drug added to an existing regimen that has toxicities.

## References

[1] Postel-Vinay S. (2015). Redefining dose-limiting toxicity. Clin. Adv. Hematol. Oncol..

[2] Gelmon K.A., Eisenhauer E.A., Harris A.L., Ratain M.J., Workman P. (1999). Anticancer agents targeting signaling molecules and cancer cell environment: challenges for drug development?. J. Natl. Cancer Inst..

[3] Sverdlov O., Wong W.K., Ryeznik Y. (2014). Adaptive clinical trial designs for phase I cancer studies. Stat. Surv..

[4] Braun T.M. (2014). The current design of oncology phase I clinical trials: progressing from algorithms to statistical models. Chin. Clin. Oncol..

[5] Zang Y., Lee J.J. (2014). Adaptive clinical trial designs in oncology. Chin. Clin. Oncol..

[6] Wong K.M., Capasso A., Eckhardt S.G. (2016). The changing landscape of phase I trials in oncology. Nat. Rev. Clin. Oncol..

[7] O'Quigley J., Pepe M., Fisher M.,L. (1990). Continual reassessment method: a practical design for phase 1 clinical trials in cancer. Biometrics.

[8] Thall P.F., Cook J.D., Estey E. (2006). Adaptive dose selection using efficacy-toxicity trade-offs: illustrations and practical considerations. J. Biopharm. Statistics.

[9] Hansen A.R., Graham D.M., Pond G.R., Siu L.L. (2014). Phase 1 trial design: is 3 + 3 the best?. Cancer control...

[10] Iasonos A., Gönen M., Bosl G.J. (2015). Scientific review of phase I protocols with novel dose-escalation designs: how much information is needed?. J. Clin. Oncol..

[11] Jaki T., Clive S., Weir C.J. (2013). Principles of dose finding studies in cancer: a comparison of trial designs. Cancer Chemother. Pharmacol..

[12] Eisenhauer E.A., O'Dwyer P.J., Christian M., Humphrey J.S. (2000). Phase I clinical trial design in cancer drug development. J. Clin. Oncol..

[13] Ji Y., Liu P., Li Y., Bekele B.N. (2010). A modified toxicity probability interval method for dose-finding trials. Clin. Trials.

[14] Blanchard M.S., Longmate J.A. (2011). Toxicity equivalence range design (TEQR): a practical Phase I design. Contemp. Clin. Trials.

[15] Liu S., Yuan Y. (2015). Bayesian optimal interval designs for phase I clinical trials. J. R. Stat. Soc. Ser. C.

[16] Babb J.S., Rogatko A. (2001). Patient specific dosing in a cancer phase I clinical trial. Stat. Med..

[17] Tighiouart M., Rogatko A. (2010). Dose finding with escalation with Overdose control (EWOC) in Cancer clinical trials. Stat. Sci..

[18] Yang S., Wang S.J., Ji Y. (2015). An integrated dose-finding tool for phase I trials in oncology. Contemp. Clin. Trials.

[19] Storer B.E. (2001). An evaluation of phase I clinical trial designs in the continuous dose–response setting. Stat. Med..

[20] Simon R., Freidlin B., Rubinstein L., Arbuck S.G., Collins J., Christian M.C. (1997). Accelerated titration designs for phase I clinical trials in oncology. J. Natl. Cancer Inst..

[21] Ivanova A. (2006). Escalation, group and A + B designs for dose-finding trials. Stat. Med..

[22] Le Tourneau C., Lee J.J., Siu L.L. (2009). Dose escalation methods in phase I cancer clinical trials. J. Natl. Cancer Inst..

[23] Liu S., Cai C., Ning J. (2013). Up-and-down designs for phase I clinical trials. Contemp. Clin. Trials.

[24] Lin Y., Shih W.J. (2001). Statistical properties of the traditional algorithm-based designs for phase I cancer clinical trials. Biostatistics.

[25] Garrett-Mayer E. (2006). The continual reassessment method for dose-finding studies: a tutorial. Clin. Trials.

[26] Lee S.M., Cheung Y.K. (2009). Model calibration in the continual reassessment method. Clin. Trials.

[27] Paoletti X., Baron B., Schöffski P., Fumoleau P., Lacombe D., Marreaud S., Sylvester R. (2006). Using the continual reassessment method: lessons learned from an EORTC phase I dose finding study. Eur. J. Cancer.

[28] Ivy S.P., Siu L.L., Garrett-Mayer E., Rubinstein L. (2010). Approaches to phase 1 clinical trial design focused on safety, efficiency, and selected patient populations: a report from the clinical trial design task force of the national cancer institute investigational drug steering committee. Clin. Cancer Res..

[29] Ivanova A., Montazer-Haghighi A., Mohanty S.G., Durham S.D. (2003). Improved up-and-down designs for phase I trials. Stat. Med..

[30] Ivanova A., Ting N. (2006). Dose-finding in oncology – nonparametric methods (chapter 4). Dose Finding in Drug Development.

[31] Le Tourneau C., Gan H.K., Razak A.R., Paoletti X. (2012). Efficiency of new dose escalation designs in dose-finding phase I trials of molecularly targeted agents. PLoS One.

